# Take your mother’s ferry: preimplantation embryo development requires maternal karyopherins for nuclear transport

**DOI:** 10.1172/JCI166279

**Published:** 2023-01-17

**Authors:** Momal Sharif, Laura Detti, Ignatia B. Van den Veyver

**Affiliations:** 1 Department of Obstetrics and Gynecology,; 2 Division of Reproductive Endocrinology and Infertility,; 3 Divisions of Maternal Fetal Medicine and Prenatal and Reproductive Genetics, and; 4 Department of Molecular and Human Genetics, Baylor College of Medicine, Houston, Texas, USA.

## Abstract

The genetic basis of preimplantation embryo arrest is slowly being unraveled. Recent discoveries point to maternally expressed proteins required for cellular functions before the embryonic genome is activated. In this issue of the *JCI*, Wang, Miyamoto, et al. suggest a critical role for karyopherin-mediated protein cargo transport between oocyte cytoplasm and nucleus. Defective maternal oocyte–expressed human karyopherin subunit *α*7 (KPNA7) and mouse KPNA2 fail to bind a critical substrate, ribosomal L1 domain-containing protein 1 (RSL1D1), affecting its transport to the nucleus. As shown in embryos of *Kpna2*-null females, the consequences are disrupted zygotic genome activation and arrest of development. These findings have important implications for diagnosis and treatment of female infertility.

## Unraveling the genetics of female infertility

Infertility affects 15% of couples (1). A myriad of male and female factors can cause infertility, and the clinical work-up for female infertility is complex, involving evaluation of ovarian and uterine function and anatomy and underlying medical disorders that can affect ability to conceive (2). The clinical genetic work-up for female infertility is still limited and often includes no more than testing for a few conditions that affect fertility, such as parental chromosome constitution or maternal carrier status for fragile X premutation, associated with premature ovarian insufficiency.

**Figure 1:**
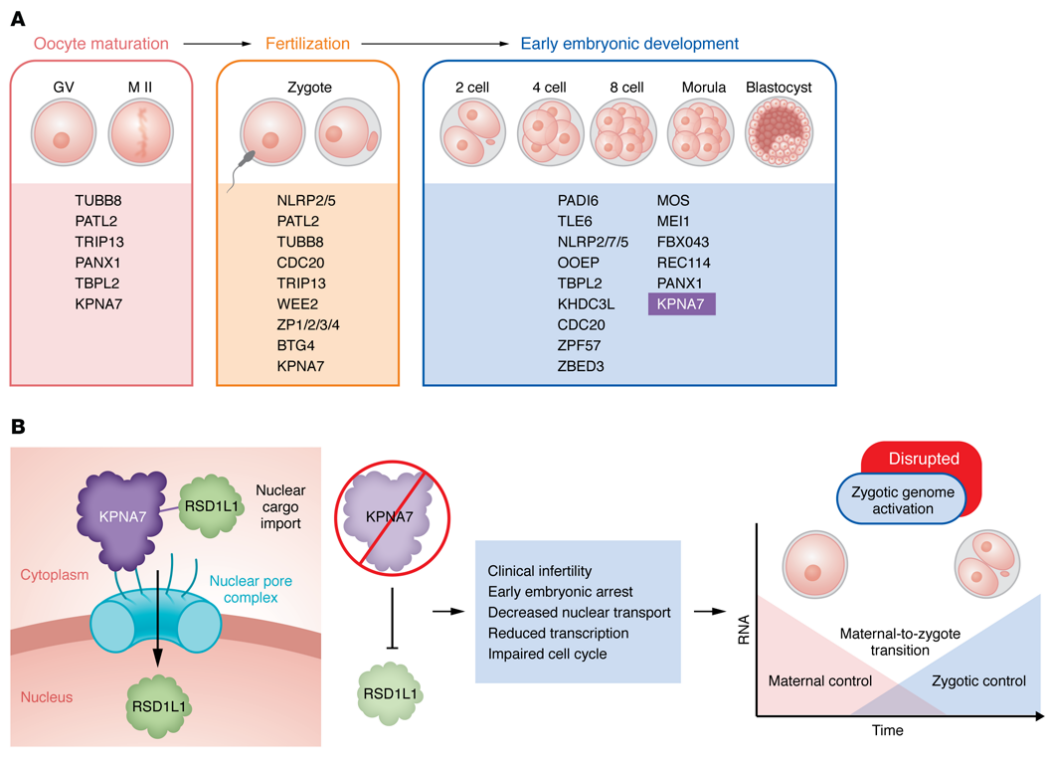
Maternal genetic factors are implicated in oocyte and early embryonic development. (**A**) There are several maternal genetic factors implicated in oocyte development, fertilization, zygotic cleavage, and early embryonic development. The genetic factors are broadly categorized based on their primary phenotypes and developmental defects. KPNA7 is implicated in preimplantation embryo arrest. (**B**) Karyopherin-mediated protein cargo transport between oocyte cytoplasm and nucleus is essential for proper ZGA. If defective, maternal oocyte–expressed human KPNA7 and its mouse homolog KPNA2 fail to bind RSD1L1, affecting its transport to the nucleus. The consequences are clinical infertility due to disrupted ZGA and early embryonic arrest (reported by Wang et al., ref. 7).

Exome and genome sequencing of DNA from cohorts of women with infertility has yielded insights into underlying genetic causes (reviewed in refs. 2–5). The implicated gene deficiencies are broadly categorized into those causing oocyte maturation defects, fertilization and zygotic defects, and preimplantation embryo arrest (Figure 1A). These genetic deficiencies explain only a small proportion of these forms of infertility, and more are yet to be discovered. Identifying these genes helps in understanding of the processes required for the transition from highly specialized, transcriptionally silent gametes to a pluripotent, rapidly dividing, early preimplantation embryo. The maternal-to-zygotic transition (MZT) depends on maternally expressed genes to facilitate meiotic cell division, spindle formation, epigenetic reprogramming, storage of maternal RNAs and proteins in mature oocytes, and their timely degradation after fertilization, when the embryo begins to transcribe its own genome (6). Factors from the nucleus, cytoplasm (in particular the oocyte subcortical maternal complex and cytoplasmic lattices), cell membrane, and zona pellucida all play a role in this transition (3).

A major cornerstone of infertility treatment involves in vitro fertilization (IVF) with short-term embryo culture (up to 5–7 days). This method allows assessment of oocyte, zygote, and embryonic phenotypes associated with unsuccessful IVF. In this issue of the *JCI*, Wang, Miyamoto, et al. (7) focus on identifying maternal genetic causes of preimplantation embryo arrest, whereby retrieved metaphase II–stage (MII-stage) oocytes appear healthy and can be fertilized, forming normal zygotes, but the embryos stop developing after one or a few cell divisions. Using exome sequencing of DNA from 606 women with this type of infertility, the authors identified deleterious compound heterozygous or homozygous variants in karyopherin subunit α7 (*KPNA7*) in ten (1.7%) families. The genetic burden of *KPNA7* matches that of *PADI6*, the most frequently implicated maternal gene in preimplantation embryo arrest (8, 9). This result revealed the requirement of another class of proteins, karyopherins, for early human embryo development (7). The finding supports the idea that nuclear shuttling of certain proteins affects development of preimplantation embryos and in particular the proper initiation of zygotic genome activation (ZGA), which requires both correctly timed degradation of maternal RNAs and proteins and activation of transcription and translation of the embryonic genome.

## Maternal karyopherin α protein family in embryonic development

A collection of maternal RNAs, proteins, and other biomolecules drives the developmental processes in early embryogenesis until the embryonic genome is activated during MZT. It is not surprising that impaired nuclear transport interrupts the intracellular trafficking of substrates that are essential for ZGA initiation. This transport is mediated by karyopherin α (also known as importin α), of which mammals encode multiple subtypes (10, 11); six have been identified in the mouse and seven in humans, referred to as karyopherin α1–α7 (KPNA1, KPNA2, KPNA3, KPNA4, KPNA5, KPNA6, and KPNA7). The transcript abundance of these seven karyopherin α’s changes substantially during development from the germinal vesicle (GV) oocyte to the embryo blastocyst stage (12), strongly suggesting that the protein family serves a critical function during embryogenesis. Among human *KPNA* genes, *KPNA7* (importin α8) is the most abundant karyopherin α in human GV and MII-stage oocytes, where DNA methylation may account for its oocyte-specific expression (13). KPNA7 remains present in early embryos, but its RNA is quickly degraded during ZGA (12, 14–16) and it becomes barely detectable in morulas and blastocysts, consistent with a role in transporting essential nuclear factors required for MZT (14). In a series of elegant experiments combining human sequence data, in vitro cell culture data, and mouse models, Wang, Miyamoto, et al. (7) first found that specific variants reduced protein levels of mutant KPNA7 and impaired binding to and transport of selected protein cargo into the nucleus. The authors identified protein RSD1L1, which is highly expressed in oocytes, as an important cargo of KPNA7 and then showed, using mutant mouse models, that *Kpna2* is the mouse homolog of human *KPNA7* (7) (Figure 1B). Maternal loss of *Kpna2* in mice affected nuclear transport of RSD1L1 and led to a phenotype of early embryo arrest with altered transcriptome at initiation of ZGA in two-cell embryos. The authors then found that maternal loss of *Rsd1l1* in mice also caused preimplantation embryo arrest, confirming the importance of RSD1L1 in this process (7). Interestingly, studies in other species had already implicated maternal karyopherin α encoded by KPNA7 homologs in early embryo development. RNAi-based knockdown experiments in bovine (14) and porcine (12) embryos showed that KPNA7 is necessary for cleavage development. *Kpna7* has also recently been described as a maternal effect gene in zebrafish (17). These data support that a conserved mechanism, important for embryonic genome activation and early development, exists across various species. Finally, Wang, Miyamoto, et al. showed that injection of *Kpna7* complementary RNA (cRNA) into maternal *Kpna2*-null mouse zygotes could reverse embryo arrest, pointing toward therapeutic possibilities for this form of early embryo arrest (7). Although exciting, considering ethical complexities, such therapy is not currently an option that can be considered in humans.

## Implications for human female infertility diagnosis and treatment

The study by Wang, Miyamoto, and colleagues adds to a growing list of maternal genes implicated in infertility caused by preimplantation embryo arrest (7). This and other studies raise the question of whether it is time for genome-wide or gene-panel sequencing for the clinical work-up of female infertility. This question is important because maternal-effect genetic causes of KPNA7-related and similar forms of infertility have a relatively low chance for effective treatment with IVF, as the primary maternal genetic defect affects all the woman’s oocytes. Whether women with infertility should be offered this genetic testing or whether it should be considered only after failed IVF with relevant oocyte or preimplantation embryo phenotypes remains an open question.

## Version 1 01/17/2023

Electronic publication
